# Severity of Dementia Is Associated with Increased Periodontal Inflamed Surface Area: Home Visit Survey of People with Cognitive Decline Living in the Community

**DOI:** 10.3390/ijerph182211961

**Published:** 2021-11-14

**Authors:** Ayako Edahiro, Tsuyoshi Okamura, Yoshiko Motohashi, Chika Takahashi, Ayami Meguro, Mika Sugiyama, Fumiko Miyamae, Tsutomu Taga, Chiaki Ura, Riko Nakayama, Mari Yamashita, Shuichi Awata

**Affiliations:** 1 Research Team for Promoting Independence and Mental Health, Tokyo Metropolitan Institute of Gerontology, Tokyo 173-0015, Japan; tokamura@tmig.or.jp (T.O.); yoshikom@tmig.or.jp (Y.M.); kuruko@tmig.or.jp (C.T.); sugiyama@center.tmig.or.jp (M.S.); fmiyamae@tmig.or.jp (F.M.); ttaga@tmig.or.jp (T.T.); ura@tmig.or.jp (C.U.); awata@tmig.or.jp (S.A.); 2 Removable Prosthodontics, Nihon University Graduate School of Dentistry at Matsudo, Chiba 271-8587, Japan; maay18015@g.nihon-u.ac.jp; 3 Department of Integrated Education and Science, Graduate School of Education, The University of Tokyo, Tokyo 113-8654, Japan; hur_jasmin@p.u-tokyo.ac.jp; 4 Research Team for Social Participation and Community Health, Tokyo Metropolitan Institute of Gerontology, Tokyo 173-0015, Japan; yama0531@tmig.or.jp

**Keywords:** people living with dementia, periodontal inflamed surface area, community-dwelling, in-home survey, interdisciplinary team, older people, Japan

## Abstract

No studies have measured the periodontal inflamed surface area in people with dementia, although periodontal disease is a major health issue in this group. This study aimed to determine the relationship between dementia severity and periodontal inflamed surface area. An interdisciplinary team, including a dentist and psychiatrist, conducted an in-home survey of older people living in the community. This cross-sectional study was designed as part of a larger cohort study. The interdisciplinary team visited 198 individuals with cognitive decline. We surveyed the clinical dementia rating, periodontal inflamed surface area, number of teeth, and other health issues. We used multiple linear regression analysis to assess the 75 people who were able to take part in all the visits. Number of teeth (Beta = 0.479, *p* < 0.001), clinical dementia rating (Beta = 0.258, *p* = 0.013), and age (Beta = 0.250, *p* = 0.017) were independently associated with periodontal inflamed surface area after adjusting for biological sex, depression, diabetes, collagen disease, visual disorder, and osteoporosis medication. To make communities more dementia-friendly, we must protect older people with dementia from developing poor oral health, which may require home visits for dental assessment.

## 1. Introduction

Oral health is an indicator of health in old age [1]. More than 50% of people aged 80 in Japan now have 20 teeth left [2,3]. This trend is also true for people with dementia. Periodontal disease is a major health issue among older people, and is a chronic inflammatory disease of the supporting tissues around the teeth [4]. It is a result of accumulation of exposure to relevant factors throughout life [5]. Ironically, it is well known that when older people have more teeth, their oral cavity is more complex, and it is harder to maintain oral cleanliness [6]. This may cause the chronic inflammation in periodontal disease, resulting in increased risk of death from aspiration pneumonia [7]. The emphasis of geriatric dentistry is shifting from merely maintaining the number of the teeth to maintaining oral function by preventing periodontal disease.

The most widely-used measure for periodontal disease is the periodontal inflamed surface area (PISA) [4]. PISA has been reported to be correlated with many medical conditions, including hypertension [7], coronary heart disease [8], diabetes [9,10], and chronic kidney disease [11,12], and is also associated with smoking [13]. However, as far as we can tell, no studies have measured PISA in people with dementia.

Oral health research among older people with cognitive impairments has mainly involved outpatients or institutional residents [14]. There is little information about community-dwelling older people with cognitive impairments [15,16]. In Japan, more than 70% of older people with low care needs and 40–50% of those with high care needs were living at home in 2020 [17]. Community-dwelling older people with dementia without access to professional dental treatment may have undetected dental problems [18]. The oral health of older people with dementia who do not visit dental outpatient clinics is overlooked by society. This may be particularly true for older people with dementia who live at home, rather than in institutions. To explore the real-world oral health problems of community-dwelling older people with dementia, we conducted a visiting survey into the oral health of community-dwelling people with cognitive decline. 

The aim of this study was to describe the distribution of PISA by dementia severity, and to determine the relationship between the two, through an in-home survey of older people living in the community by an interdisciplinary team including a dentist and psychiatrist.

## 2. Materials and Methods

### 2.1. Study Population and Design

**Figure 1:**
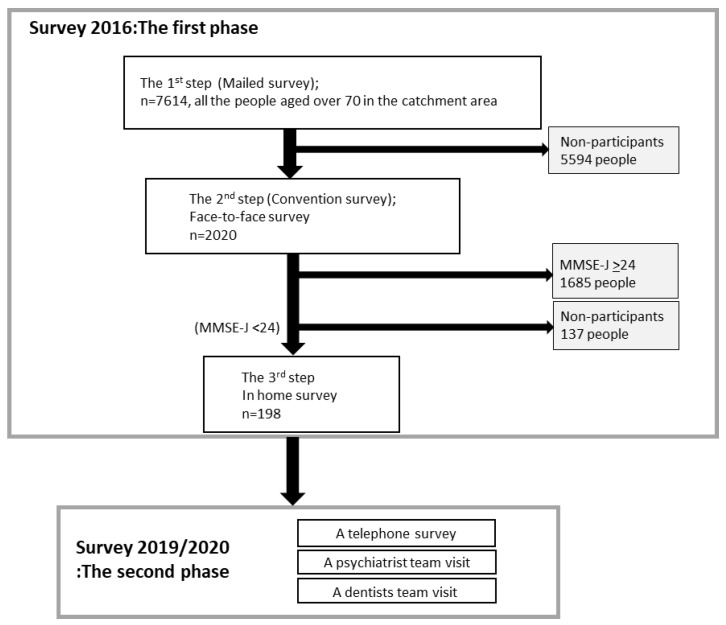
Study flow. The Takashimadaira Study included a 2016 epidemiological study (the first phase) to study the prevalence of dementia and a 2019 prognostic study (the second phase) among those at high risk of cognitive decline and social isolation.

This cross-sectional study was designed as part of a larger cohort study, the Takashimadaira Study. The Takashimadaira Study included (1) an epidemiological study in 2016 (the first phase) to identify people with cognitive impairment, and (2) a follow-up study in 2019 (the second phase) (Figure 1).

In the first phase, questionnaires were mailed to 7614 residents over 70 years old in the target area of Tokyo, and 5430 were returned. A total of 2020 people then participated in a face-to-face survey that included the Mini Mental State Examination (Japanese version) (MMSE-J). Finally, 198 of the 335 people who scored MMSE-J < 24 were visited by the interdisciplinary team, including a psychiatrist [19].

In the second phase, we carried out a telephone survey of the 198 individuals with cognitive decline who received a visiting survey in 2016 [20]. A psychiatrist team and a dentist team also conducted a home visit survey in 2019/2020. Unfortunately, because of COVID–19, only 75 people were able to complete all the visits and were therefore eligible for this study. The Supplementary Table S1 shows the scores in the first phase for participants who did and did not participate in the second phase (Supplementary Table S1). Study participants in the second phase showed higher cognitive function and less impairment caused by dementia. In addition, 18 participants passed away between the phases.

This study was approved by the Ethics Committee of the Tokyo Metropolitan Institute of Gerontology (2016-2559-33, 2019-1924-18, 2019-3146-36), and was conducted in accordance with the Strengthening the Reporting of Observational Studies in Epidemiology Statement [21].

### 2.2. Measurements

#### 2.2.1. Sociodemographic Status Obtained in the First Phase (2016)

The sociodemographic data were collected through questionnaires in the first phase (2016) and verified during visits. They included age, biological sex, economic status, years of education, residential status, and other diseases. Economic status was divided into “economically distressed”, “normal”, and “comfortable”. Residential status was divided into “living alone” and “sharing accommodation, including with family”.

#### 2.2.2. Cognitive and Psychological Assessment 

Cognitive assessment included the Japanese version of the Mini Mental State Examination (MMSE-J) [22,23], the Dementia Assessment Sheet for Community-based Integrated Care System (21 items) (DASC-21), and the clinical dementia rating (CDR) [24], which were assessed in the first and second phases. The CDR is widely used as the gold standard method for assessing dementia [25]. Using the CDR scale, the severity of dementia was classified as no dementia (CDR0), questionable dementia (CDR0.5), mild dementia (CDR1), moderate dementia (CDR2) or severe dementia (CDR3). All tests were performed by a single psychiatrist during the home visit survey in 2019/2020. The diagnosis of dementia was based on Diagnostic and Statistical Manual of Mental Disorders, 4th edition (DSM-IV) criteria [26]. The psychiatrist also used the two-question screen for depression [27] and the Japanese version of the World Health Organization’s Five Well-Being Index (WHO-5-J) [28,29] to assess depression and psychological well-being.

#### 2.2.3. Dental Assessment

Dental assessment was conducted in the second phase. A certified geriatric dentist brought adequate artificial lighting to the participants’ homes. The participants lay supine on a bed or sofa and underwent an oral examination. The dentist carried out a comprehensive periodontal examination, including:

A. Number of teeth. To measure the area of inflammation around the root of the tooth in periodontal disease, the number of teeth were counted, including current teeth (teeth able to participate in mastication), retained roots and heavily decayed teeth. Dental implants were not included in the count.

B. The PISA and the periodontal epithelical surface area (PESA). A periodontal probe WHO (YDM, Tokyo, Japan) was used to measure the clinical attachment level (CAL), the probing pocket depth (PPD) and gingival recession in millimeters at six sites on the roots of all teeth. The CAL was measured as the distance from the cemento-enamel junction (CEJ) to the bottom of the pocket. Where the CEJ was obscured by the edge of a crown restoration, its location was estimated from the adjacent landmarks and the anatomy of the tooth. In teeth without gingival proliferation, the recession was calculated by subtracting the PPD from the CAL. Bleeding on probing (BOP) was measured at the point of bleeding after withdrawal of the probe. PESA was calculated beforehand, and PISA was calculated using PPD, recession, and BOP, to reflect the inflammatory state of periodontal tissues, using the method of Nesse W2008 [4]. PISA is known to be correlated with the number of teeth, CAL and BOP [30]. BOP was included in the study as percentage of bleeding on probing.

C. The Simplified Oral Hygiene Index (OHI-S). To assess oral hygiene, we calculated the Debris Index (DI), Calculus Index (CI) and Oral Hygiene Index (OHI-S) set out by Greene and Vermillion [31].

D. Oral function. We used the repetitive saliva swallowing test (RSST) to assess swallowing function, this is a screening test for swallowing function, where participants are asked to repeatedly swallow their own saliva. It shows the latency of the swallowing reflex in effortful swallowing. The number of seconds between the instruction to start swallowing and the first swallow was given as the RSSTfirst, and the number of swallows in 30 seconds was the RSST30 [32].

#### 2.2.4. Other Measures

Medication was identified by reviewing the most recent medication records in the second phase. We included osteoporosis medication or injections, antihypertensive medication, diabetes medication or injective agents, and anti-thrombotic therapy. Blood pressure was measured by the dentist at the time of the visit. A mini nutritional assessment (MNA) was made during the home visit to assess nutritional status [33]. Living habits included drinking habits, smoking, oral health behavior, and eating sweet food including the presence of candy and sweet drinks. Smoking was calculated by daily consumption multiplied by years of smoking, as the exposure dose, using the Brinkman Index [34]. We assessed three aspects: oral health self-care behavior; professional oral care (POC) within the previous year, which included oral health instruction, professional tooth cleaning, and tongue cleaning; and whether participants had attended a dental consultation within the previous year. Oral health self-care behavior was classified into: (1) before sleep, (2) after meals, (3) before meals, and (4) sometimes do not care. All visits were conducted in the presence of family members, who confirmed the participants’ daily activities. 

#### 2.2.5. Statistical Analysis

The CDR was divided into three groups (0, 0.5–1 and 2–3) and examined using one-way analysis of variance with Bonferroni’s post-hoc test or the χ^2^ test to describe the distribution of PISA and dementia severity. Pearson’s correlation coefficients were used to evaluate the correlations between factors associated with PISA. In addition, Spearman’s rank correlation coefficients were used to analyze CDR data. Potential confounders were selected from the factors correlated with PISA as covariates in a multivariate model for PISA. We used the directed acyclic graph method [35] to construct the final multiple linear regression model. In creating the directed acyclic graph, we used factors that have been previously re-ported to be correlated with PISA and dementia to create a causal diagram (see the Supplementary Figure S1) [7,8,13,36]. We chose the number of present teeth as a representative value of oral status and excluded BOP (%), DI, CI, OHI-S and RSST from the final model because of collinearity. Using this process, our model minimized the bias in the empirical results. The final model was adjusted for age, biological sex, depression, diabetes, collagen disease, visual disorder, osteoporosis medication, and number of teeth. The multiple linear regression analysis used a stepwise method, with a *p*-value of < 0.05 considered statistically significant. We used IBM SPSS version 27 (IBM Corp., Armonk, NY, USA). This was a secondary study of the Takashimadaira Study, and therefore, no prior sample size calculations were performed.

## 3. Results

### 3.1. Characteristics of Participants by Dementia Severity

The mean scores for PISA and other covariates for each category of CDR are shown in Table 1. The CDR 2–3 group had significantly higher PISA (*p* < 0.001), BOP (*p* < 0.001) and DI (*p* = 0.010). Their oropharyngeal function was significantly impaired, as shown by RSSTfirst (*p* < 0.001). The CDR 2–3 group had more years of education (*p* = 0.018), were less likely to live alone (*p* = 0.002), were more likely to have diabetes (*p* = 0.015) and had worse nutritional status (*p* = 0.012). They were also less likely to have good oral health self-care habits (*p* = 0.001) and to have experienced POC or a dental visit within the previous year (*p* = 0.023).

### 3.2. Comparison of Each Factor to PISA and Dementia Severity

The results of the correlation analysis are shown in Table 2. Number of teeth (Pearson’s r = 0.447), DI (r = 0.378), CI (r = 0.361), OHI-S (r = 0.379), RSSTfirst (r = 0.453), rheumatoid collagen disease (r = 0.402), DASC-21 (r = 0.353) and CDR (r = 0.436) were all moderately correlated with PISA. Depression (r = 0.233), diabetes (r = 0.220), visual disorder (r = 0.242) and oral health self-care habits (r = 0.235) were weakly correlated with PISA.

### 3.3. Factors Associated with Increased PISA

The results of multiple linear regression analysis for PISA are shown in Table 3. The multiple linear regression analysis for PISA showed that number of teeth (Beta = 0.479, *p* < 0.001), CDR (Beta = 0.258, *p* = 0.013) and age (Beta = 0.250, *p* = 0.017) were all independently associated with PISA after adjusting for biological sex, depression, diabetes, collagen disease, visual disorder, and medication for osteoporosis. Older people with moderate to severe dementia and more teeth were more likely to be living with chronic inflammation in their oral cavity.

## 4. Discussion

To the best of our knowledge, this is the first study to compare PISA in people with dementia living in the community by severity of dementia. Previous studies have emphasized the difficulty of obtaining accurate diagnoses of both dementia and chronic periodontitis [37]. The strength of this study is that it was conducted by an interdisciplinary team involving both a psychiatrist and dentists, using a precise definition. We clearly showed that age and severity of dementia were associated with PISA, even after adjusting for the number of teeth, suggesting that severity of dementia is closely asssociated with increased PISA.

It is often difficult to recognize the dental care needs of people with dementia living in densely populated urban areas [38]. Older people receiving psychiatric care are reported to have higher dental care needs [39]. Participants in this study also had more teeth than other reports of older people with dementia in Japan [40]. Older people with more remaining teeth, subjective chewing and no oral pain are therefore less likely to see a dentist [41]. Periodontal disease is generally painless, and thus people with dementia who have periodontal disease tend not to go to the dentist, and may therefore have unmet oral care needs.

The mean age of the study population was 83.5 ± 5.3 years, making it a relatively old population. It has been suggested that *Porphyromonas gingivalis*, which causes periodontal disease, may vary within patients, leading to an inefficient or ineffective immune response, particularly in older populations [42]. The phenomenon of aging itself may also influence the host response to bacterial infection and contribute to the inflammatory process [43].

The relationship between periodontal disease and dementia is assumed to be cyclical and reciprocal, rather than unidirectional. Some hypotheses suggest that periodontal disease accelerates the development of dementia. One previous study hypothesized that brain invasion of *P. gingivalis* and lipopolysaccharides may be linked to inflammatory mediators in the blood, enhancing the permeability of the blood–brain barrier, and enabling them to penetrate the brain [44]. It has been reported that PISA in young adults affects the onset of mild cognitive impairment [45].

Several reasons for worsening oral hygiene in dementia and cognitive impairment have been reported. Visuospatial cognitive decline may make mouth cleaning difficult [46]. People with dementia may also lose interest in habits and/or have reduced manual dexterity, making cleaning harder. Social barriers may prevent people with dementia from seeking help for oral problems [18]. Additionally, people with dementia and oral frailty often favor a soft, adherent, carbohydrate-based diet [47]. This makes the remaining teeth more susceptible to harm.

We suggest that periodontal disease worsens in people with decreasing cognitive function as their dementia becomes more severe. First, older people in the prodromal stage may experience social isolation and apathy [48], leading to worsening of periodontal disease. Second, people with cognitive impairment have changed CAL because of poor oral hygiene during the prodromal stage [49]. Third, their increased difficulty with oral self-management leads to increased periodontal bacteria and gingival bleeding, resulting in a larger PISA. Fourth, over time after the onset of dementia, PISA becomes worse because of neglected oral hygiene, unless teeth are lost. Thus, we speculate that the time since onset of dementia is related to higher PISA.

Older people with dementia living in the community may continue with inadequate oral self-care and not seek dental care because their dementia progresses slowly. They may miss the opportunity to change how they care for their oral health. Even if their oral self-care ability decreases, people often do not seek help with oral care. They tend to have difficulties accessing dental clinics, with particular barriers to preventive and regular dental visits [50]. Untreated oral disease has serious consequences, and the decision to see a dentist should not be based solely on self-reported symptoms and distress, especially for older people with dementia. In addition, PISA was reported to be associated with mild cognitive impairment [45], and was found to be associated with advanced dementia in older adults in the current study. There is a clear need to support early and ongoing efforts to maintain oral health in older people. Dementia-friendly communities are now a global goal in many areas [51]. It is also necessary to spread the use of technology that allows older people with advanced dementia to control oral inflammation without stress [52]. People with dementia and social isolation may also need special accommodations from society. Positive social interventions such as home-visiting dental assessment teams might be a first step in this process.

This study had some limitations. First, the number of participants was limited because we invited people with cognitive decline living in the community who were being followed in a long-term longitudinal study. The effect sizes in the statistical analyses in the current study were limited because of the number of participants affected by the COVID-19 pandemic. Older people with dementia who live at home and not in institutions generally have no medical factors that could worsen their life expectancy and receive either family support or public care support. The study therefore did not include people with serious cerebrovascular disease, Parkinson’s disease or renal disease requiring dialysis. The CDR2-3 moderate and severe dementia group who maintained community living was too small to be a representative group. It is, therefore, impossible to make a simple comparison with institutionalized or hospitalized older people with dementia of the same age. Second, the study was conducted at participants’ home and therefore involved no medical tests such as blood tests or imaging tests. Third, inflammatory markers and periodontal species were not identified, and thus, it is impossible to discuss specific species in interpreting the results. Fourth, the content of self-reported survey responses by older people with dementia may contain inaccuracies. Although it is difficult to conduct home visit dental surveys with the participation of older people who continue living at home in the community even if their dementia has progressed, it will be necessary to examine this issue in more detail in future studies. Finally, this study was cross-sectional, so we were unable to examine any causal relations. A case-controlled longitudinal study is needed to clarify this.

## 5. Conclusions

In this study, we clearly showed that age and severity of dementia were associated with PISA, even after adjusting for the number of teeth. This suggests that severity of dementia is closely associated with increased PISA. We suggest more oral intervention is essential for community-dwelling older people with dementia, who often experience barriers to preventive and regular dental visits. To move toward dementia-friendly communities, we must protect older people with dementia from developing poor oral health.

## Figures and Tables

**Table 1:** Oral health status, sociodemographic status, and clinical and behavioral characteristics stratified by CDR groups.

	CDR 0 N = 36	CDR 0.5–1 N = 30	CDR 2–3 N = 9	Total N = 75	One Way ANOVA with Bonferroni Post-Hoc Test/χ^2^ Test	
	Ave ± SD, (%)	Ave ± SD, (%)	Ave ± SD, (%)	Ave ± SD, (%)	*p*-Value	
Oral health status						
PISA	98.8 ± 122.2	165.7 ± 149.6	412.6 ± 419.0	163.2 ± 210.7	<0.001	CDR 0 < CDR 2–3, CDR 0.5–1 < CDR 2–3
No. of teeth (including decayed teeth)	14.4 ± 9.3	16.0 ± 8.0	17.4 ± 10.0	15.4 ± 8.8	0.579	
PESA	527.6 ± 391.0	688.2 ± 357.6	911.0 ± 590.8	637.9 ± 419.7	0.032	CDR 0 < CDR 2–3
BOP (%)	0.1 ± 0.1	0.2 ± 0.1	0.3 ± 0.2	0.1 ± 0.1	<0.001	CDR 0 < CDR 2–3, CDR 0.5–1 < CDR 2–3
Debris Index	2.9 ± 1.6	4.0 ± 1.6	4.2 ± 1.7	3.5 ± 1.7	0.010	CDR 0 < CDR 0.5–1
Calculus Index	2.5 ± 1.5	3.1 ± 1.5	3.2 ± 1.9	2.8 ± 1.5	0.315	
OHI-S	5.7 ± 2.5	7.1 ± 2.6	7.9 ± 2.3	6.6 ± 2.6	0.034	
RSST first (1st, min)	1.3 ± 0.5	2.0 ± 2.0	5.8 ± 7.1	2.1 ± 2.9	<0.001	CDR 0 < CDR 2–3, CDR 0.5–1 < CDR 2–3
RSST (time/min)	4.3 ± 1.4	3.8 ± 1.1	2.9 ± 1.2	3.9 ± 1.4	0.026	CDR 0 < CDR 2–3
Sociodemographic status						
Biological sex (women %)	50.0%	76.7%	44.4%	60.0%	0.053	
Age	81.7 ± 5.0	86.1 ± 5.3	82.2±2.8	83.5 ± 5.3	0.002	CDR 0 < CDR 0.5–1
Education (years)	10.1 ± 2.2	11.1 ± 2.5	12.6±2.8	10.8 ± 2.5	0.018	CDR 0 < CDR 2–3
Economic status						
Economically distressed	57.6%	10.3%	22.2%	33.8%	0.002	
Normal	36.4%	69.0%	66.7%	53.5%		
Comfortable	6.1%	20.7%	11.1%	12.7%		
Living alone	38.9%	63.3%	0.0%	44.0%	0.002	
Clinical status						
Other diseases						
Hypertension	61.1%	83.3%	44.4%	68.0%	0.042	
Cerebrovascular disease	5.6%	3.3%	0.0%	4.0%	0.727	
Cardiovascular disorders	30.6%	26.7%	22.2%	28.0%	0.864	
Diabetes	25.7%	10.0%	55.6%	23.0%	0.015	
Hyperlipidemia	47.2%	40.0%	0.0%	38.7%	0.033	
Renal disease	13.9%	3.3%	0.0%	8.0%	0.186	
Collagen disease (rheumatoid arthritis)	2.8%	0.0%	11.1%	2.7%	0.192	
Visual disorder	41.7%	66.7%	42.9%	52.1%	0.113	
Blood pressure						
Systolic blood pressure Diastolic blood pressure	153.1 ± 26.7	154.8 ± 22.5	146.8 ± 33.8	153.0 ± 25.8	0.726	
	87.8 ± 17.1	88.0 ± 21.5	87.7 ± 24.9	87.8 ± 19.6	0.999	
Medication						
Osteoporosis	19.4%	30.0%	22.2%	24.0%	0.601	
Hypertension	58.3%	73.3%	77.8%	66.7%	0.329	
Diabetes	22.2%	10.0%	44.4%	20.0%	0.069	
Anti-thrombotic	19.4%	33.3%	33.3%	26.7%	0.397	
Cognitive Assessment						
DASC-21	23.3 ± 4.1	30.2 ± 9.0	61.8 ± 10.5	30.8 ± 14.2	< 0.001	CDR 0 < CDR 0.5–1, CDR 0 < CDR 2–3, CDR 0.5–1 < CDR 2–3
MMSE-J	24.1 ± 2.7	21.1 ± 3.0	12.3 ± 3.6	21.5 ± 4.7	< 0.001	CDR 0 > CDR 0.5–1, CDR 0 > CDR 2–3, CDR 0.5–1 > CDR 2–3
Mental health status						
Depression (two questions)	25.0%	10.0%	22.2%	18.7%	0.285	
WHO-5-J	16.4 ± 6.5	15.5 ± 6.0	15.1 ± 4.7	15.9 ± 6.1	0.771	
Living and behavioral status						
Alcohol						
Habitual drinker	25.0%	30.0%	22.2%	26.7%	0.855	
Stopped drinking	27.8%	20.0%	22.2%	24.0%	0.756	
Smoking						
Brinkman index	519.9 ± 608.8	252.9 ± 305.3	530.0 ± 449.7	450.1 ± 524.7	0.479	
Nutrition (MNA®)	24.8 ± 3.6	24.2 ± 3.1	20.8 ± 4.0	24.1 ± 3.6	0.012	CDR 0 > CDR 2–3, CDR 0.5–1 > CDR 2–3
Sweet food behavior	19.4%	13.3%	22.2%	17.3%	0.742	
Oral health self-care behavior						
Before sleep	75.0%	60.0%	33.3%	64.0%	0.001	
After meals	25.0%	26.7%	11.1%	24.0%		
Before meals	0.0%	6.7%	11.1%	4.0%		
Sometimes do not care	0.0%	6.7%	44.4%	8.0%		
POC within previous year (yes)	63.9%	36.7%	22.2%	48.0%	0.023	
Dental consultation within previous year (yes)	58.3%	46.7%	44.4%	52.0%	0.569	

The distribution of PISA and each item by dementia severity were examined by one-way analysis of variance (ANOVA) with Bonferroni’s post-hoc test or χ^2^ test. CDR: clinical dementia rating; PISA: periodontal inflamed surface area; PESA: periodontal epithelical surface area; BOP: bleeding on probing; OHI-S: Oral Hygiene Index; RSST: Repetitive Saliva Swallowing Test; DASC-21: Dementia Assessment Sheet in Community-based Integrated Care System (21 items) (21–84); MMSE-J: Mini Mental State Examination—Japanese version (0–30); WHO-5-J: Japanese version of the World Health Organization—Five Well-Being Index (0–25); MNA: Mini Nutritional Assessment (0–30); POC: professional oral care, including oral health instruction, professional tooth cleaning, and tongue cleaning.

**Table 2:** The correlation of factors associated with PISA and CDR.

	Pearson’s r		Spearman’s ρ	
	PISA		CDR	
Oral health status				
Number of teeth (including decayed teeth)	0.447	**	0.119	
PISA			0.331	**
BOP (%)	0.866	**	0.385	**
Debris Index	0.378	**	0.396	**
Calculus Index	0.361	**	0.203	
OHI-S	0.379	**	0.357	**
RSSTfirst (1st, min)	0.453	**	0.308	**
RSST (time/min)	−0.141		−0.273	*
Sociodemographic status				
Biological sex	0.016		0.125	
Age	0.115		0.253	
Education (years)	0.007		0.347	**
Economic status	0.021		0.408	**
Living alone	−0.015		−0.023	
Clinical status				
Concomitant disease				
Hypertension	−0.114		0.047	
Cerebrovascular disease	−0.117		−0.100	
Cardiovascular disorders	0.057		−0.086	
Diabetes	0.220	*	0.043	
Hyperlipidemia	−0.129		−0.235	*
Renal disease	−0.094		−0.215	
Collagen disease (rheumatoid arthritis)	0.402	**	0.054	
Visual disorder	0.242	*	0.127	
Blood pressure				
Systolic blood pressure	−0.107		−0.073	
Diastolic blood pressure	−0.126		−0.022	
Medication				
Osteoporosis	0.227	*	0.068	
Hypertension	0.076		0.150	
Diabetes	0.006		0.029	
Anti-thrombotic	0.126		0.129	
Cognitive assessment				
DASC-21	0.353	**	0.737	**
MMSE-J	−0.222	*	−0.671	**
CDR	0.436	**		
Mental health status				
Depression (two questions)	0.233	*	−0.140	
WHO-5-J; Mental health	−0.009		−0.118	
Living and behavioral status				
Alcohol: habitual drinker/stopped drinking/never drank	0.218		0.030	
Smoking: Brinkman index	0.071		−0.073	
Nutrition: MNA	−0.189		−0.258	*
Sweet food behavior	0.135		−0.032	
Oral health self-care behavior	0.235	*	0.284	*
POC within the previous year	0.079		0.280	*
Dental consultation within the previous year	0.155		0.129	

Pearson’s correlation coefficients were used to evaluate the correlations between PISA and each factor. Spearman’s rank correlation coefficients were used to analyze CDR data. ** correlation is significant at the 0.01 level; * correlation is significant at the 0.05 level. CDR: clinical dementia rating; PISA: periodontal inflamed surface area; PESA: periodontal epithelical surface area; BOP: bleeding on probing; OHI-S: Oral Hygiene Index; RSST: Repetitive Saliva Swallowing Test; DASC-21: Dementia Assessment Sheet in Community-based Integrated Care System (21 items) (21–84); MMSE-J: Mini Mental State Examination—Japanese version (0–30); WHO-5-J: Japanese version of the World Health Organization—Five Well-Being Index (0–25); MNA: Mini Nutritional Assessment (0–30); POC: professional oral care, including oral health instruction, professional tooth cleaning, and tongue cleaning.

**Table 3:** Multiple linear regression analysis for PISA: the final multivariate model.

	Unstandardized Coefficient		Standardized Coefficient	t	*p*-Value	Tolerance	VIF
	B	Std. Error	Beta				
(Constant)	−694.662	277.898		−2.500	0.015		
No. of teeth (including decayed teeth)	9.219	1.957	0.479	4.711	< 0.001	0.983	1.018
CDR	62.721	24.642	0.258	2.545	0.013	0.987	1.013
Age	8.003	3.276	0.250	2.443	0.017	0.973	1.028

Determinants of PISA were identified using stepwise multiple linear regression analysis where independent variables with the least significance (*p* > 0.05) were sequentially omitted. Adjusted for age, biological sex, depression, diabetes, collagen disease, visual disorder, medication for osteoporosis, number of teeth and CDR. R^2^ = 0.350, adjusted R^2^ = 0.320. VIF: variance inflation factor; CDR: Clinical Dementia Rating; PISA: periodontal inflamed surface area.

## Data Availability

The data that support the findings of this study are available from the corresponding author upon reasonable request. The data are not publicly available because of ethical and legal restrictions imposed by the Ethics Committee of the Tokyo Metropolitan Institute of Gerontology.

## Supplementary Materials

The following are available online at https://www.mdpi.com/article/10.3390/ijerph182211961/s1, Supplementary Table S1: comparison of demographic and cognitive measures between participants who did and did not participate in the second phase. Supplementary Figure S1: the directed acyclic graph with CDR as an exposure factor and PISA as an outcome.
