# Supplementary material for: Severity of Dementia Is Associated with Increased Periodontal Inflamed Surface Area: Home Visit Survey of People with Cognitive Decline Living in the Community
Source: Int J Environ Res Public Health. 2021 Nov 14;18(22):11961. doi: 10.3390/ijerph182211961 (PMC8618461; doi:10.3390/ijerph182211961)
Supplement: Supplementary file 1 [file ijerph-18-11961-s001.zip › ijerph-1396266-supplementary.pdf]

Table S1. Comparison of demographic and cognitive measures between participants who did and did not participate in the second phase.

		Participants N = 75		Non-participants N = 123		Total N = 198		P-value
		Ave ± SD	Range (%)	Ave ± SD	Range (%)	Ave ± SD	Range (%)	
Scores of the first phase	Biological sex (women %)		60.0%		59.3%		59.6%	0.524
	Age	80.5 ± 5.3	70–95	81.4 ± 6.3	70–99	81.0 ± 6.0	70–99	0.339
	MMSE-J	21.0 ± 2.1	13–23	19.5 ± 4.2	3–23	20.0 ± 3.6	3–23	0.003
	DASC-21	25.7 ± 8.0	21–63	30.3 ± 12.6	21–80	28.6 ± 11.3	21–80	0.005
Outcomes over 3 years	Died before the second phase (%)		0.0%		14.6%		9.1%	

The table shows a comparison of the scores in the first phase between participants who did and did not participate in the second phase.

Biological sex in the first stage was examined using χ^2 tests. Age and the results of cognitive assessment in the first stage were analyzed using t-tests. The percentage of participants who had died before reaching the second phase was also shown.

DASC-21: Dementia Assessment Sheet in Community-based Integrated Care System (21 items) (21–84); MMSE-J: Mini Mental State Examination – Japanese (0–30)

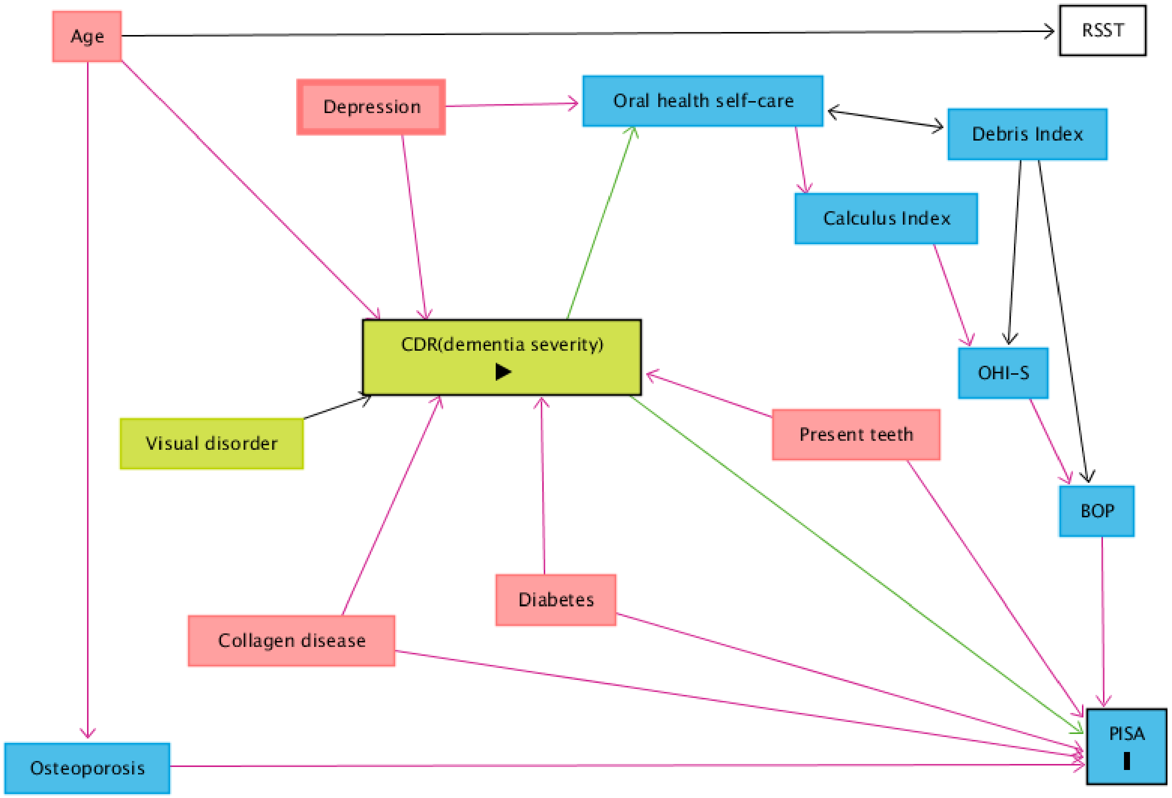

Supplemental figure: The directed acyclic graph with CDR as an exposure factor and PISA as an outcome.
